# Systems Analysis of ATF3 in Stress Response and Cancer Reveals Opposing Effects on Pro-Apoptotic Genes in p53 Pathway

**DOI:** 10.1371/journal.pone.0026848

**Published:** 2011-10-26

**Authors:** Yujiro Tanaka, Aya Nakamura, Masaki Suimye Morioka, Shoko Inoue, Mimi Tamamori-Adachi, Kazuhiko Yamada, Kenji Taketani, Junya Kawauchi, Miki Tanaka-Okamoto, Jun Miyoshi, Hiroshi Tanaka, Shigetaka Kitajima

**Affiliations:** 1 Laboratory of Genome Structure and Regulation, School of Biomedical Science and Department of Biochemical Genetics, Medical Research Institute, Tokyo Medical and Dental University, Tokyo, Japan; 2 Department of Computational Biology, School of Medical Sciences and Department of Bioinformatics, Tokyo Medical and Dental University, Tokyo, Japan; 3 Department of Molecular Biology, Osaka Medical Center for Cancer and Cardiovascular Diseases, Osaka, Japan; Wayne State University School of Medicine, United States of America

## Abstract

Stress-inducible transcription factors play a pivotal role in cellular adaptation to environment to maintain homeostasis and integrity of the genome. Activating transcription factor 3 (ATF3) is induced by a variety of stress and inflammatory conditions and is over-expressed in many kinds of cancer cells. However, molecular mechanisms underlying pleiotropic functions of ATF3 have remained elusive. Here we employed systems analysis to identify genome-wide targets of ATF3 that is either induced by an alkylating agent methyl methanesulfonate (MMS) or over-expressed in a prostate tumour cell line LNCaP. We show that stress-induced and cancer-associated ATF3 is recruited to 5,984 and 1,423 targets, respectively, in the human genome, 89% of which are common. Notably, ATF3 targets are highly enriched for not only ATF/CRE motifs but also binding sites of several other stress-inducible transcription factors indicating an extensive network of stress response factors in transcriptional regulation of target genes. Further analysis of effects of ATF3 knockdown on these targets revealed that stress-induced ATF3 regulates genes in metabolic pathways, cell cycle, apoptosis, cell adhesion, and signalling including insulin, p53, Wnt, and VEGF pathways. Cancer-associated ATF3 is involved in regulation of distinct sets of genes in processes such as calcium signalling, Wnt, p53 and diabetes pathways. Notably, stress-induced ATF3 binds to 40% of p53 targets and activates pro-apoptotic genes such as TNFRSF10B/DR5 and BBC3/PUMA. Cancer-associated ATF3, by contrast, represses these pro-apoptotic genes in addition to CDKN1A/p21. Taken together, our data reveal an extensive network of stress-inducible transcription factors and demonstrate that ATF3 has opposing, cell context-dependent effects on p53 target genes in DNA damage response and cancer development.

## Introduction

Transcription factors play important roles in temporal regulation of gene expression in serum stimulation of human cells[1], [2]. Cellular adaptation to various environmental stress conditions is also regulated by transcription factors that co-ordinately modulate expression of genes involved in maintenance of cellular homeostasis and genetic integrity. Such a system plays an important role for not only survival of normal cells but also resistance of cancer cells to metabolic and genotoxic stresses. A crucial step towards understanding molecular mechanisms underlying stress responses is the identification of target genes of each transcription factor. Studies in yeast employing gene expression profiling [3], [4] and more recently systems analysis by chromatin immunoprecipitation of transcription factors [5] have revealed a genome-wide networks of transcription factors regulating expression of genes that orchestrate cell cycle, gene transcription, protein synthesis, and DNA repair in response to MMS. In mammals, the tumour suppressor p53 plays a pivotal role in DNA damage response through transcriptional control of several hundred genes [6], [7]. Importantly, only a small subset of p53 targets are activated under specific conditions[8], indicating that either binding of p53 to targets [9]–[11] or trans-activation potential of p53 proteins [12], [13] may be affected by accessory molecules.

ATF3 is a member of the ATF/CREB family of basic-leucine zipper (b-Zip) type transcription factors [14]and is a highly versatile stress sensor for a wide range of conditions including hypoxia, hyponutrition, oxidative stresses, ER stresses, and various genotoxic stresses[15], [16] as well as inflammatory reactions [17], [18]. ATF3 is also activated by serum stimulation and downstream of c-Myc[19], and is frequently over-expressed in various tumours including those of the prostate[20], breast[21], and Hodgkin's lymphomas[22]. Importantly, several lines of evidence has indicated a close link between ATF3 and p53 signalling pathways. Thus, ATF3 is induced downstream of p53 upon DNA damage and functions as an effector of p53-mediated cell death [6], [23]–[25]. In addition, ATF3 potentiates p53 by directly binding and inhibiting its ubiquitilation, implying that ATF3 can modulate the activity of p53 [26], [27]. Furthermore, ATF3 appears to confer a negative feedback to the p53 pathway by down-regulating *TP53* gene expression[28], recapitulating a similar feedback regulation of inflammatory cytokine genes by ATF3[17]. Corroborating such a negative feedback model, a recent study has demonstrated that ATF3 is induced by Cyclosporin, an immune suppressor, and promotes skin cancer by down-regulating *TP53* [29]. Taken together, these studies suggest that ATF3 interacts with the p53 pathway both as a downstream effector of p53-mediated cell death and as a positive and negative regulator of p53 signalling.

Alteration in interaction between ATF3 and transcription factors such as p53 in different type of cells and/or environmental conditions may in part account for distinct effects of ATF3 on cell fate (i.e. pro-apoptotic or growth-promoting for untransformed cells or malignantly transformed cells, respectively)[21]. Previous work from our laboratory has also described pleiotropic functions of ATF3 under various stress conditions[18], [19], [25], [28], [30]. Here we carried out systems analysis of ATF3 targets and identified thousands of ATF3 binding sites in the genome. We show that ATF3 constitutes an extensively overlapping gene regulatory network with other stress-inducible transcription factors and regulates cell cycle, cell death, adhesion, and several signalling pathways including p53. Notably, ATF3 binds to 40% of known targets of p53 and regulates apoptotic cell death through co-activation of a subset of pro-apoptotic genes stress response while repressing the same targets in cancer cells constitutively expressing ATF3. Possible switching mechanisms between pro-survival and pro-apoptotic ATF3 functions will be discussed.

## Results

### Systems analysis identifies thousands of ATF3 targets in the human genome

**Figure 1:**
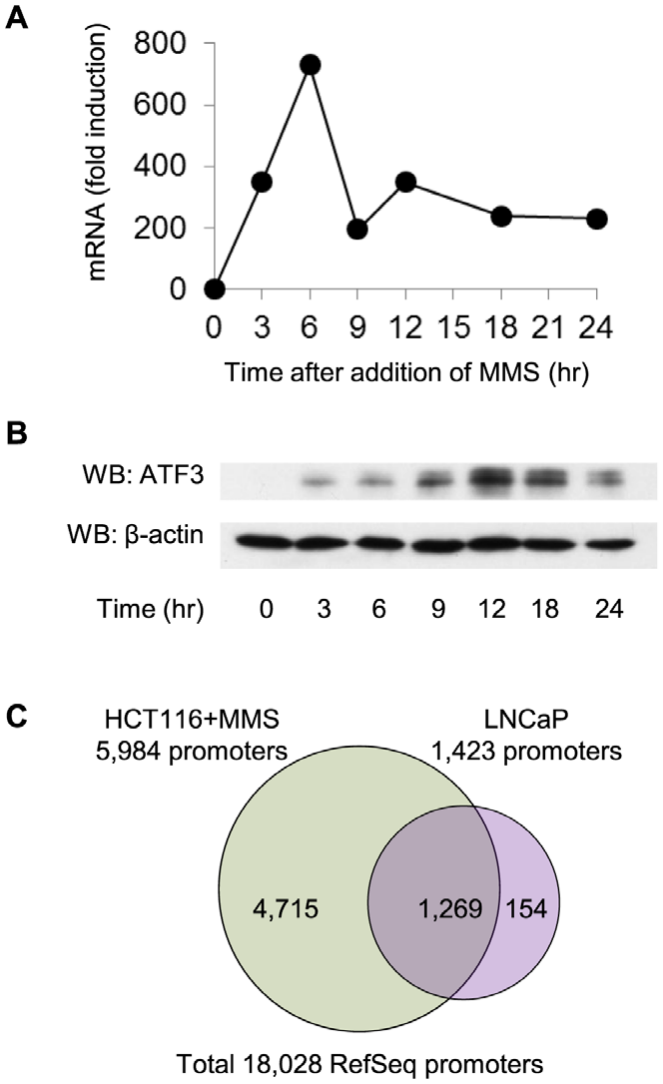
ChIP-on-chip analysis of ATF3 targets. (A) RT-PCR analysis of ATF3 in HCT116 cells treated with 50 ng/ml MMS. (B) Western blot analysis of ATF3 proteins in MMS-treated HCT116 cells. (C)Common and unique targets of ATF3 in HCT116 cells and LNCaP cells.

To identify genomic targets of ATF3, chromatin immunoprecipitation analysis was carried out using HCT116 human colon cancer cell line stimulated by MMS and LNCaP prostate cancer cell line which constitutively expresses ATF3. As reported previously[24], MMS treatment of HCT116 cells induced transient expression of ATF3 reaching a peak at 6 hours after stimulation (Fig. 1A), whereas increased levels of ATF3 proteins were detected after 3 hours and reached a maximum level at 12 hours of stimulation (Fig. 1B). Genomic DNA was prepared from either HCT116 cells treated with MMS for 6 hours or untreated LNCaP cells, immunoprecipitated with anti-ATF3 antibodies, and hybridized to NimbleGen's human RefSeq HG18 promoter tiling arrays. Subsequently, peak detection was carried out by Model-based Analysis of 2-colour Arrays (MA2C) package[31], which revealed an unexpectedly large number of ATF3 targets at a cut-off of 0.2% FDR (Fig. S1A and S1B). We identified 5,984 and 1,423 targets of ATF3 in MMS-treated HCT116 cells and LNCaP cells, respectively, 1,269 (i.e. 89%) of which were sheared between the two models. (Fig. 1C). We detected no targets on the Y chromosome from HCT116 consistent with its female origin. We also successfully identified thirteen ATF3 targets which had been previously shown to be regulated by ATF3 in various cell types.

### Motifs of stress-associated transcription factors are over-represented in ATF3 targets

**Figure 2:**
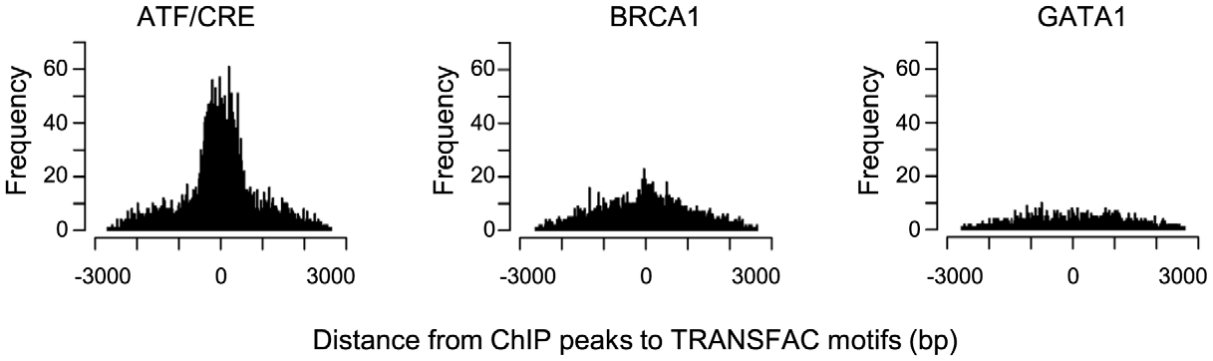
Physical distance between predicted motifs and ATF3 peaks. Distribution of physical distance between ATF/CRE, BRCA1, and GATA1 motifs on ATF3 targets from ATF3 peaks identified by ChIP analysis. ATF3 peaks largely coincide with ATF/CRE motifs but show no association with unrelated BRCA1 and GATA1 motifs.

ATF3 could be recruited to its targets either by directly binding to a consensus recognition sequence TGACGTCA or alternatively by interacting with other transcription factors including a large number of b-Zip proteins[14], [32]. To assess if the ATF/CRE motif or any other transcription factor motifs are enriched among the potential ATF3 targets, the number of promoters in the ATF3 targets containing at least one hit of a given motif in the TRANSFAC database was analysed by the P-Match algorithm (Fig. S2). Table 1 summarizes enrichment of a given motif as represented by the number of promoters in ATF3 targets containing the motif subtracted by the number of promoters in a background gene set containing the same motif. As expected, ATF/CRE binding sites were the most enriched motifs in ATF3 targets (*p*-value for HCT116-specific targets: 3.76×10^−40^ to 4.34×10^−22^, *p*-value for common targets of HCT116 and LNCaP cells: 0 to 4.20×10^−6^). Moreover, physical distance between predicted ATF/CRE sequences of the motif scan and ATF3 peaks of the ChIP analysis was within a range of a few hundred base pairs (i.e. close to the resolution of the ChIP analysis) for the majority of ATF3 targets (Fig. 2). By contrast, there was no such association between unrelated BRCA1 and GATA1 motifs and ATF3 peaks (Fig. 2). Taken together, these data strongly suggest that the majority of ATF3 peaks of the ChIP analysis can be explained by direct binding of ATF3 to ATF/CRE motifs.

**Table 1:** Motif scan of ATF3 targets.

Binding factor	Matrix	HCT-116*	Common*	LNCaP*	Function/Response
CREB/ATF family	V$CREB_02	309	96		ATF3 binding
	V$CREB_Q3	290	218		
	V$CREBATF_Q6	290	141		
	V$TAXCREB_01	153	53		
	V$TAXCREB_02		49		
CREB/ATF family	V$AP1_Q2_01	40	40	14	bZip (jun/fos) binding
	V$AP1_Q4_01	50	47		
CHOP/DDIT3	V$CHOP_01	58	60		bZip, ER stress
HIF-1	V$AHR_Q5	169	60		Hypoxia, DNA damage
	V$AHRARNT_01	139	61		
	V$AHRHIF_Q6	132	76		
	V$ARNT_01	53	30		
	V$HIF1_Q3	73			
NF-Y	V$NFY_01	196	16		DNA damage
	V$NFY_Q6_01	197	27		
E2F	V$E2F_03	299	119		DNA damage
	V$E2F_Q6_01	261	103		
c-Ets	V$CETS1P54_01	157	108		Sheer stress, hypoxia, reactive oxygen
	V$CETS1P54_02	85	55		
	V$CETS1P54_03		95		
	V$ETS_Q6		32		
YY1/INO80S/NF-E1	V$YY1_01	44	61		ER stress, DNA damage
	V$YY1_Q6				
USF2	V$USF2_Q6	20	73		bZip, UV irradiation
RFX1	V$EFC_Q6		107		HU, UV irradiation
	V$RFX_Q6		44		
EGR-1	V$EGR1_01	147			DNA damage
	V$KROX_Q6	107			
FOXJ2	V$FOXJ2_02	132			DNA damage
Sp1	V$SP1_Q6	127			DNA damage, interact with ATF3

List of motifs in TRANSFAC database enriched in ATF3 targets. Those targets which are unique to HCT116 cells, unique to LNCaP cells, or common between them are indicated with the number promoters with at least one hit of a given motif subtracted by the number of promoters with the same motif in a background gene set.

*Difference in the number of promoters with the motif between the test and background gene sets.

Intriguingly, the motif scan also revealed that binding sites of other stress-inducible transcription factors were highly over-represented in ATF3 targets in response to DNA damage (Table 1). These included major regulators of ER stress (DDIT3, NF-E1), hypoxia (HIF-1A), UV stress (USF2, RFX1), oxidative stress (c-Ets), and DNA damage (NF-Y, E2F, EGR-1, FOXJ2, Sp1). Of note, members of the b-Zip family (AP-1, DDIT3, and USF2) have a potential to heterodimerize with ATF3[33] and Sp1 has been reported to physically interact with ATF3[34]. It is therefore possible that ATF3 is recruited to a subset of its targets indirectly through association with other stress-inducible transcription factors.

### ATF3 regulates distinct biological processes in stress response and in cancer

**Figure 3:**
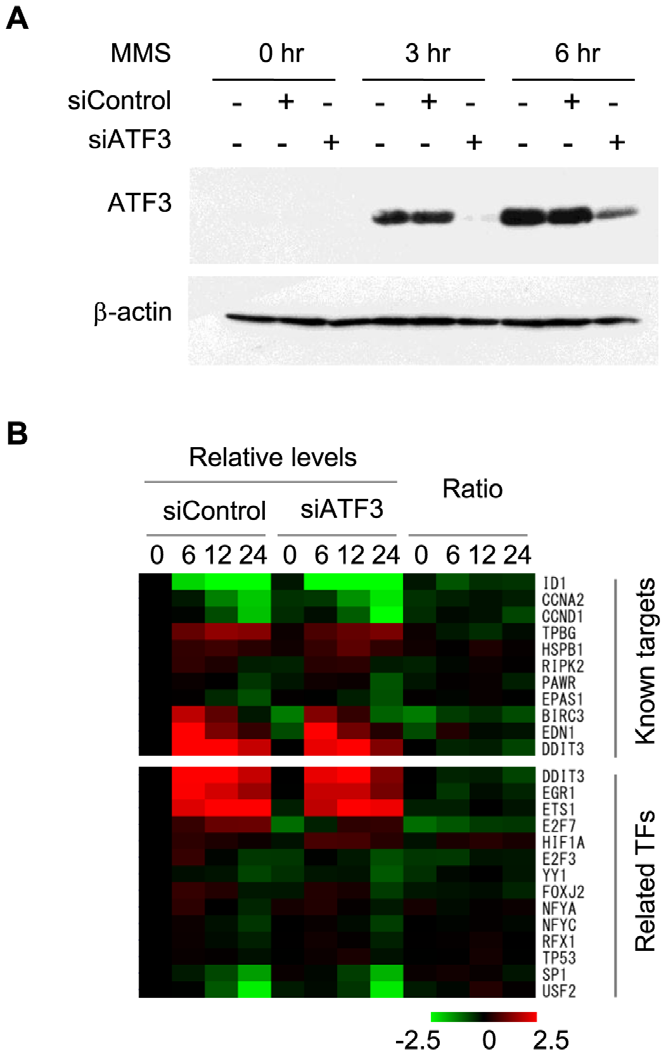
Impact of ATF3 knockdown on target gene expression. (A) HCT116 cells were transfected with either siRNA for ATF3 (siATF3) or scrambled siRNA (siControl) and treated with MMS for 0, 3, and 6 hours. Amount of ATF3 proteins was analysed by Western blot with β-actin as a loading control. MMS induces ATF3 at 3 and 6 hours after stimulation, which is significantly blocked by siATF3. (B) Heat map representation of gene expression levels at 0, 6, 12, and 24 hours after MMS stimulation for known targets of ATF3 (upper panel) or transcription factors which binding motifs are over-represented in ATF3 targets (lower panel). Signals are normalized against those at time 0 of siControl and relative levels or ratio between siATF3/siControl are colour coded from 2^−2.5^ to 2^2.5^.

Having established potential targets of ATF3 in the genome, next we aimed to identify those genes which expression is regulated by ATF3. To this end, we assessed the impact of ATF3 knockdown by siRNA (Fig. 3A) on gene expression patterns at 0, 6, 12, and 24 hours after stimulation by MMS using Agilent Whole Human Genome Microarray. After normalization of arrays using a group of house keeping genes (Fig. S3A), two thirds of the genes (i.e. 28,872 probes out of 43,925) were found to be expressed above background levels in HCT116 cells (Fig. S3B). By contrast, 90% of the ATF3 targets were expressed above background levels, suggesting that ATF3 preferentially associates with actively transcribed genes. At a global level, comparison of signals (log_2_ ratio) between control and knockdown cells revealed a limited impact of ATF3 down-regulation on transcriptome (Fig. S3B and S3C).

Further analysis of the ChIP-identified targets of ATF3 indicated that expression of 6–30% genes were affected by ATF3 knockdown below 0.8-fold or above 1.2-fold at different time points after MMS stimulation. To identify biological processes regulated by ATF3, pathway mapping was carried out using DAVID[35] for a subset of ATF3 targets either down- or up-regulated by ATF3 knockdown (modified Fisher's exact test *p*-value <0.1). As summarized in Table 2, stress-induced ATF3 was associated with diverse cellular processes such as metabolic pathways (sugars, amino acids, lipid), anabolic and catabolic processes (steroid and folate biosynthesis, ubiquitilation, urea cycle), energy generation (TCA cycle, oxidative phosphorylation), cell cycle, apoptosis, adhesion, cytoskeleton, and signalling pathways (ErbB, p53, insulin, Wnt, VEGF).

**Table 2:** BioCarta pathway mapping of ATF3 targets.

Genes down-regulated by ATF3 knockdown					
BioCarta pathway	p-value	0 hr	6 hr	12 hr	24 hr
h_atrbrcaPathway:Role of BRCA1, BRCA2 and ATR in Cancer Susceptibility	0.0471	5			
h_caspasePathway:Caspase Cascade in Apoptosis	0.0594		4		
h_cellcyclePathway:Cyclins and Cell Cycle Regulation	0.00142				6
h_cxcr4Pathway:CXCR4 Signaling Pathway	0.00874	6			
h_d4gdiPathway:D4-GDI Signaling Pathway	0.0842		3		
h_deathPathway:Induction of apoptosis through DR3 and DR4/5 Death Receptors	0.094		4		
h_ecmPathway:Erk and PI-3 Kinase Are Necessary for Collagen Binding in Corneal Epithelia	0.0477		4		
h_eif4Pathway:Regulation of eIF4e and p70 S6 Kinase	0.0471	5			
h_g1Pathway:Cell Cycle: G1/S Check Point	0.0776	5			
	0.00302				6
h_gpcrPathway:Signaling Pathway from G-Protein Families	0.0471	5			
h_gsk3Pathway:Inactivation of Gsk3 by AKT causes accumulation of b-catenin in Alveolar Macrophages	0.0776	5			
h_HivnefPathway:HIV-I Nef: negative effector of Fas and TNF	0.0572		6		
h_igf1mtorpathway:Skeletal muscle hypertrophy is regulated via AKT/mTOR pathway	0.0349	5			
h_metPathway:Signaling of Hepatocyte Growth Factor Receptor	0.00348	8			
h_nfatPathway:NFAT and Hypertrophy of the heart (Transcription in the broken heart)	0.0771	6			
h_nthiPathway:NFkB activation by Nontypeable Hemophilus influenzae	0.0149		5		
h_pmlPathway:Regulation of transcriptional activity by PML	0.0106	5			
h_ptenPathway:PTEN dependent cell cycle arrest and apoptosis	0.0296	5			
	0.0372		4		
h_RacCycDPathway:Influence of Ras and Rho proteins on G1 to S Transition	0.0152	6			
	0.0685				4
h_sppaPathway:Aspirin Blocks Signaling Pathway Involved in Platelet Activation	0.0296	5			
h_stressPathway:TNF/Stress Related Signaling	0.0793		4		

Genes up-regulated by ATF3 knockdown					
BioCarta pathway	p-value	0 hr	6 hr	12 hr	24 hr
h_egfPathway:EGF Signaling Pathway	0.067	5			
	0.00201		4		
h_igf1Pathway:IGF-1 Signaling Pathway	0.0169		3		
h_il2rbPathway:IL-2 Receptor Beta Chain in T cell Activation	0.0918				2
h_il6Pathway:IL 6 signalling pathway	0.024	5			
	0.000781		4		
h_insulinPathway:Insulin Signaling Pathway	0.0186		3		
h_pcafpathway:The information-processing pathway at the IFN-beta enhancer	0.0796	3			
h_pdgfPathway:PDGF Signaling Pathway	0.0594	5			
	0.00179		4		
h_plcePathway:Phospholipase C-epsilon pathwa	0.0796	3			
	0.0795		2		
	0.0504			2	
h_setPathway:Granzyme A mediated Apoptosis Pathway	0.0783	4			
h_telPathway:Telomeres, Telomerase, Cellular Aging, and Immortality	0.0529				2
h_tercPathway:Overview of telomerase RNA component gene hTerc Transcriptional Regulation	0.0461	3			
h_TPOPathway:TPO Signaling Pathway	0.0153		3		
h_vegfPathway:VEGF, Hypoxia, and Angiogenesis	0.0571	4			

Gene sets either down-regulated below 0.8-fold (A) or up-regulated above 1.2-fold (B) by ATF3 knockdown in MMS-treated HCT116 cells were subjected to KEGG pathway mapping using DAVID[35]. Those pathways with *p*-value <0.1 are listed with the number of genes affected at different time points after MMS stimulation.

Next, to identify potential targets of ATF3 in cancer-associated ATF3, we carried out ATF3 knockdown and expression profiling using LNCaP cells (Fig. S4). Pathway mapping analysis of gene subsets either down-regulated (<0.8-fold) or up-regulated (>1.2-fold) by knockdown of ATF3 is summarized in Table 3. Constitutively expressed ATF3 in LNCaP cells appeared to be involved in biological processes rather different from those in stress responses including cell adhesion, diabetes mellitus, and signalling (ErbB, p53, Wnt, calcium) with little association with metabolic processes. Taken together, these data indicate that metabolic pathways, cell cycle, apoptosis, and insulin and VEGF signalling are unique targets of stress-induced ATF3, whereas diabetes mellitus and calcium signalling, but not cell cycle and apoptosis, are unique targets of ATF3 expressed in cancer cells. The potential link between ATF3 and diabetes pathway as found in our study is consistent with a recent study showing that ATF3 activated in obese adipose cells contribute to insulin resistance through down-regulation of adiponectin and a glucose transporter GLUT4[36].

**Table 3:** Pathway mapping of ATF3 targets in cancer.

Genes down-regulated by ATF3 knockdown		
KEGG pathway	p-value	Count
hsa00340:Histidine metabolism	5.88.E-02	11
hsa01510:Neurodegenerative Diseases	1.76.E-02	12
hsa02010:ABC transporters - General	9.95.E-02	11
hsa04012:ErbB signaling pathway	1.47.E-02	21
hsa04020:Calcium signaling pathway	3.23.E-02	35
hsa04310:Wnt signaling pathway	2.12.E-02	32
hsa04360:Axon guidance	5.72.E-05	37
hsa04510:Focal adhesion	5.04.E-06	53
hsa04512:ECM-receptor interaction	5.11.E-03	23
hsa04520:Adherens junction	2.85.E-03	21
hsa04614:Renin-angiotensin system	2.55.E-02	7
hsa04640:Hematopoietic cell lineage	5.27.E-02	19
hsa04660:T cell receptor signaling pathway	7.08.E-02	20
hsa04810:Regulation of actin cytoskeleton	3.60.E-04	50
hsa04916:Melanogenesis	3.42.E-02	22
hsa05010:Alzheimer's disease	1.29.E-02	10
hsa05210:Colorectal cancer	9.12.E-02	18
hsa05212:Pancreatic cancer	9.00.E-02	16
hsa05214:Glioma	7.82.E-02	14
hsa05215:Prostate cancer	1.89.E-02	21
hsa05217:Basal cell carcinoma	9.46.E-02	13
hsa05220:Chronic myeloid leukemia	1.15.E-03	22
hsa05222:Small cell lung cancer	8.21.E-04	25

Genes up-regulated by ATF3 knockdown		
KEEG pathway	p-value	Count
hsa00220:Urea cycle and metabolism of amino groups	8.71.E-02	7
hsa04020:Calcium signaling pathway	3.82.E-02	27
hsa04060:Cytokine-cytokine receptor interaction	3.18.E-03	42
hsa04080:Neuroactive ligand-receptor interaction	8.24.E-03	40
hsa04115:p53 signaling pathway	4.79.E-02	13
hsa04350:TGF-beta signaling pathway	8.40.E-02	15
hsa04930:Type II diabetes mellitus	1.59.E-02	11
hsa04940:Type I diabetes mellitus	1.63.E-03	12
hsa04950:Maturity onset diabetes of the young	2.96.E-03	9

Gene sets either down-regulated below 0.8-fold (A) or up-regulated above 1.2-fold (B) by ATF3 knockdown in LNCaP cells were subjected to KEGG pathway mapping using DAVID[35]. Those pathways with *p*-value <0.1 are listed with the number of genes affected.

### DNA damage-induced ATF3 activates a subset of pro-apoptotic genes of the p53 pathway

**Figure 4:**
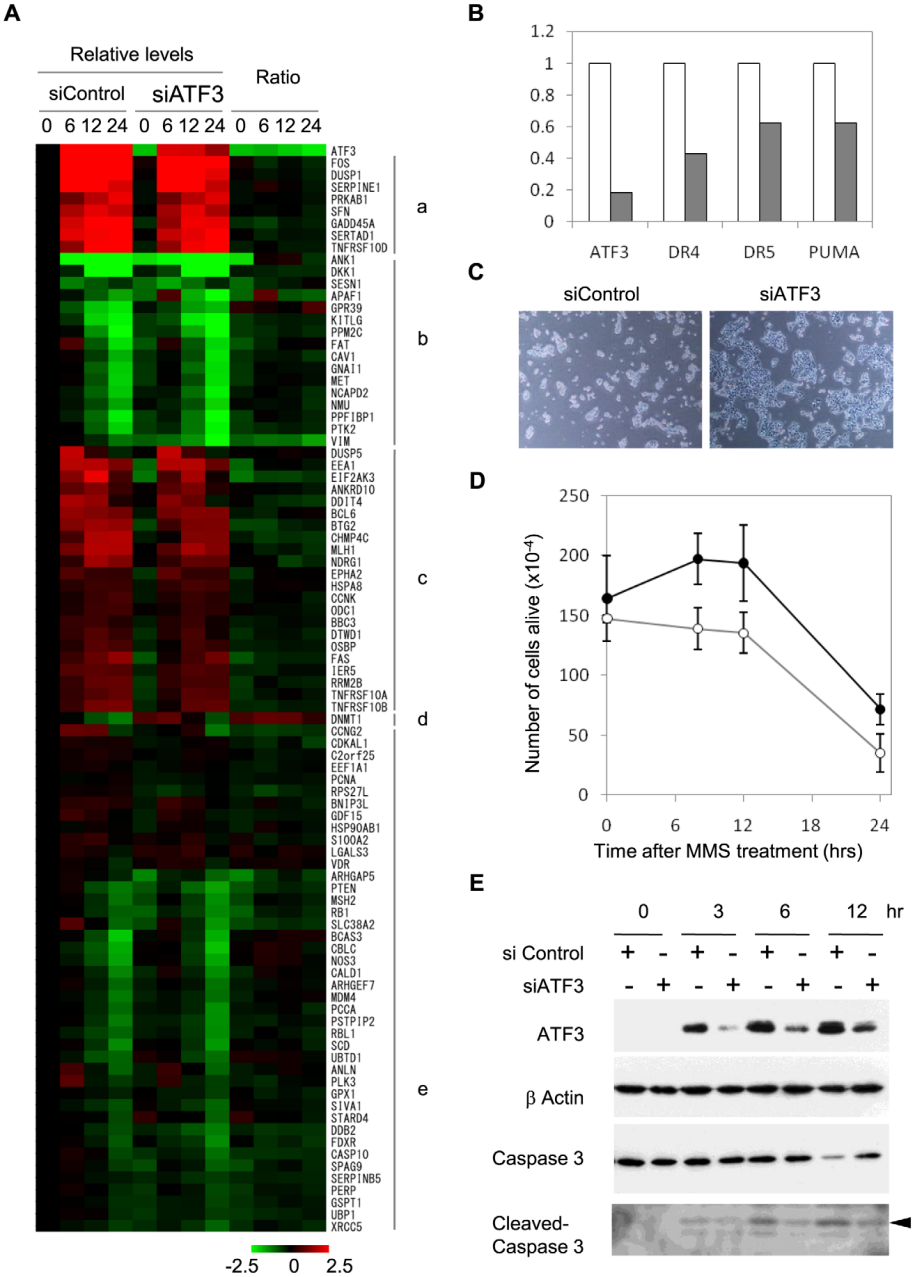
Regulation of p53 target genes by ATF3 in DNA damage response. (A) Heat map representation of gene expression levels at 0, 6, 12, and 24 hours after MMS stimulation for common targets of ATF3 and p53. Signals are normalized against those at time 0 of siControl and relative levels or ratio between siATF3/siControl are colour coded from 2^-2.5^ to 2^2.5^. Genes are clustered according to expression patterns after MMS stimulation: a, strongly activated; b, strongly repressed, c, weakly activated; d, mixed; e, weakly repressed. Most genes are weakly down-regulated by ATF3 knockdown, while DNMT1 is an exception that is up-regulated by ATF3 knockdown. (B) Quantitative RT-PCR analysis of ATF3 and pro-apoptotic targets of p53. Expression levels in siATF3-transfected cells (hashed bars) are indicated relative to those in control (open bars). (C) Phase contrast micrograph of HCT116 cells after treatment with 50 ng/ml MMS for 12 hours in the presence of siControl or siATF3. (D) Number of live HCT116 cells after treatment with 50 ng/ml MMS for 0, 8, 12, and 24 hours in the presence of siControl (open circles) or siATF3 (closed circles). (E) Activation of Caspase 3 as measured by its cleaved products. HCT116 cells were transfected with siControl or siATF3 and treated with 50 ng/ml MMS for 0, 3, 6, and 12 hours. Whole cell extracts were subjected to Western blot analysis using antibodies against ATF3, β-actin, Caspase 3, and cleaved Caspase 3. Arrowhead indicates cleaved Caspase 3 of the predicted molecular weight.

Identification of p53 pathway as a potential target of ATF3 prompted us to further analyse regulation of p53 target genes by ATF3. First we analysed how many of previously known p53 targets[6] are also targets of ATF3 in our ChIP assay. We found that stress-induced ATF3 was recruited to as many as 40% (i.e. 97/244) of p53 targets, implying for the first time that a large fraction of p53 targets may be transcriptionally co-regulated by ATF3. Next, we assessed the impact of ATF3 knockdown on expression of the common targets of ATF3 and p53 at various time points after stimulation of HCT116 cells by MMS. As illustrated by heat map in Fig. 4A (ratio of signals in siControl and siATF3), most of them are slightly down-regulated by ATF3 knockdown including pro-apoptotic genes such as BBC3/PUMA, TNFRSF10A/DR4 and TNFRSF10B/DR5.

**Figure 5:**
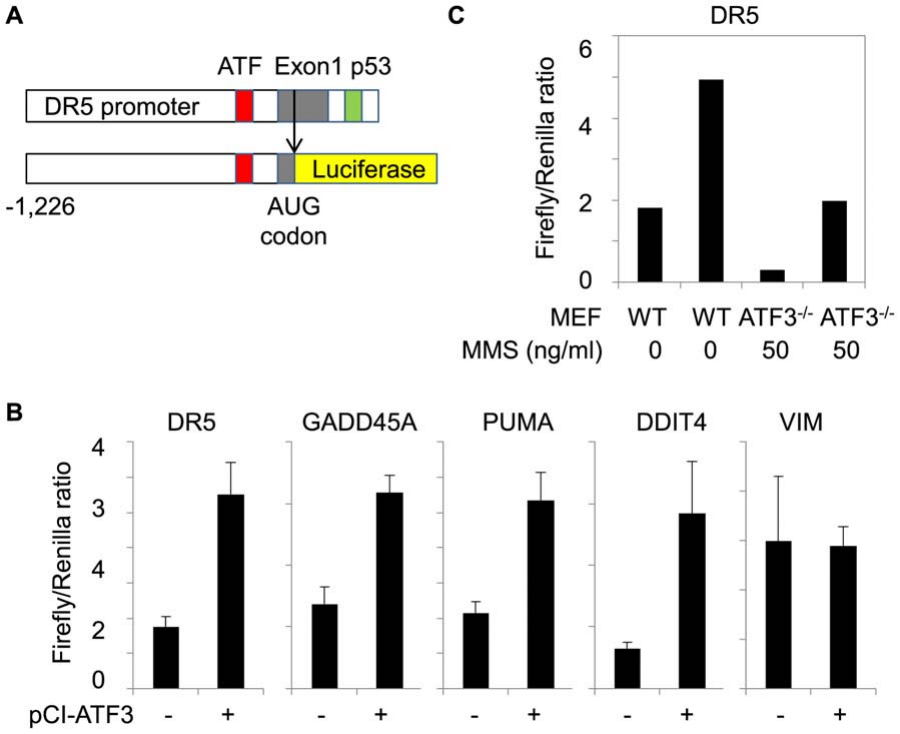
Activation of p53 target genes by ATF3. (A) The DR5-luciferase reporter construct containing a DR5 promoter fragment from −1,226 (PstI site) to +1 (AUG codon) which was subcloned into PicaGene PGV-B2. ATF/CRE motifs (ATF) and p53 motif (p53) are indicated. (B) Dual luciferase reporter assays for TNFRSF10B/DR5, GADD45A, BBC3/PUMA, DDIT4, and VIM. HCT116 cells were transfected with each reporter and stimulated with 50 ng/ml MMS for 12 hours. Firefly luciferase activity was normalized by CMV-renilla luciferase activity. (C) Dual luciferase assay using wildtype or ATF3^-/-^ mouse embryonic fibroblasts (MEF).

To further assess the function of ATF3 in p53 target gene expression, we carried out quantitative RT-PCR analysis of pro-apoptotic genes in MMS-stimulated cells pre-transfected with either control siRNA or siATF3. As shown in Fig. 4B, ATF3 knockdown caused significantly reduced induction of TNFRSF10A/DR4, TNFRSF10B/DR5, and BBC3/PUMA by MMS treatment. Next we analysed effects of ectopically expressed ATF3 on p53 target genes using luciferase reporters harbouring proximal promoters with an ATF/CRE motif as depicted in Fig. 5A. Co-transfection of HCT116 cells with luciferase reporters and ATF3 expression vectors resulted in significant activation of TNFRSF10B/DR5, GADD45A, BBC3/PUMA, and DDIT4 (Fig. 5B), consistent with microarray data showing that ATF3 knockdown caused attenuated expression of these genes (Fig. 4A). By contrast, the vimentin gene, which was repressed by MMS treatment (Fig. 4A), was not significantly activated by ectopic expression of ATF3 (Fig. 5B).

To elucidate the function of ATF3 *in vivo*, we took advantage of ATF3-deficient mice recently established in our laboratory ([37]). Mouse embryonic fibroblasts were transfected with DR5-luciferase reporters and stimulated with MMS. Interestingly, loss of ATF3 caused significantly reduced levels of basal DR5 promoter activity as shown in Fig. 5C, suggesting that basal DR5 expression was dependent on ATF3. Moreover, MMS-induced activation of DR5 promoter was reduced approximately by half in ATF3^-/-^ cells as compared to wildtype cells, implying that DNA damage-induced activation of DR5 was partially dependent on ATF3. Taken together, these loss- and gain-of-function studies demonstrate that ATF3 activates select targets of p53.

### DNA damage-induced ATF3 sensitizes cells to cell death

To assess biological significance of cross-talk between ATF3 and p53, we next analysed the effect of ATF3 knockdown on cell viability after stimulation by MMS. As depicted in Fig. 4C and 4D, there were a larger number of HCT116 cells transfected with siATF3 than those transfected with control siRNA after MMS treatment. Such difference in cell number could reflect either increased cell growth or decreased cell death. To address this issue, we analysed activation of Caspase 3, a hallmark of apoptotic cell death, by Western blot. As depicted in Fig. 4E, knockdown of ATF3 caused reduction in activation of Caspase 3 as judged by the production of cleaved Caspase 3. These data are consistent with ATF3-mediated activation of pro-apoptotic genes (Fig. 4B, Fig. 5) and strongly suggest that DNA damage-induced ATF3 contributes to stress-induced cell death by co-activating a subset of p53 target genes.

### Cancer-associated ATF3 promotes cell proliferation and inhibits apoptosis

**Figure 6:**
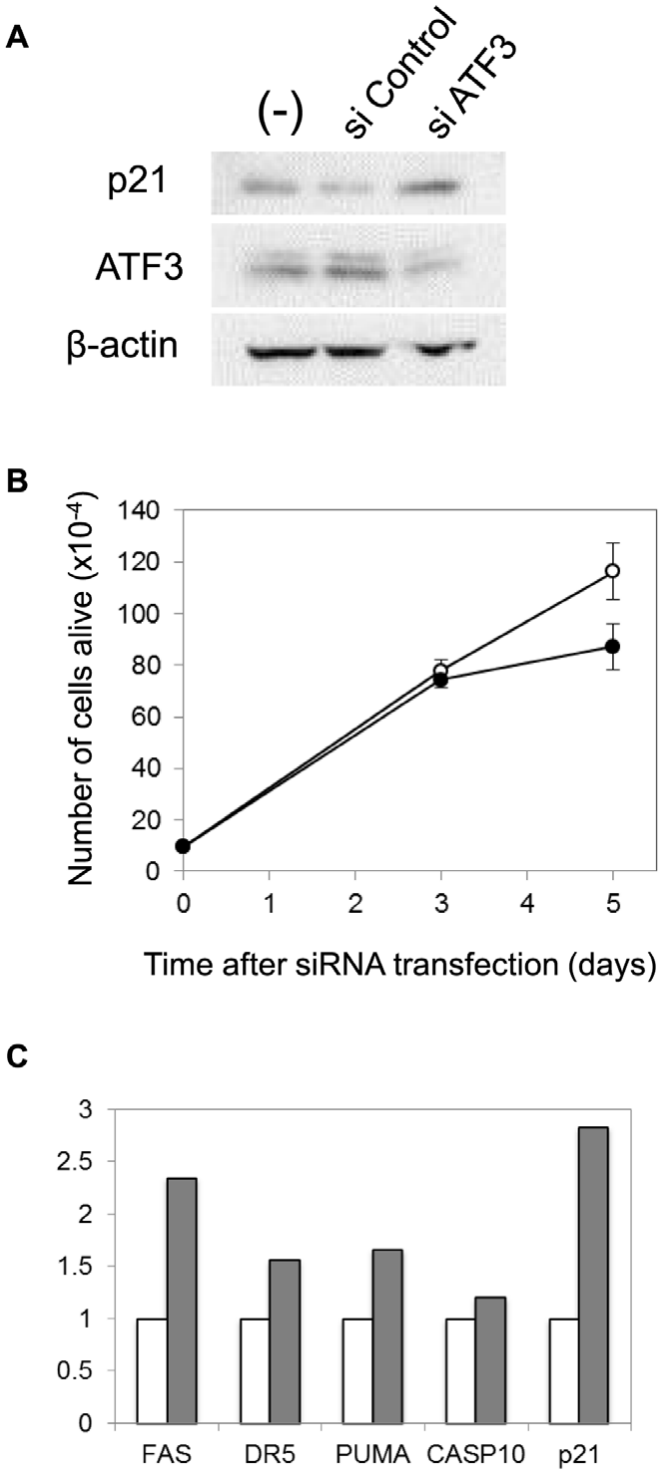
Regulation of p53 target genes by ATF3 in cancer. (A) Western blot analysis of ATF3 and p21 in LNCaP cells transfected with siControl or siATF3 with β-actin as a loading control. (B) Number of viable cells at 0, 3, and 5 days after transfection with siControl (open circles) or siATF3 (closed circles). (C) Quantitative RT-PCR analysis of pro-apoptotic genes in p53 pathway and p21 after transfection. Levels of expression in siATF3 (hashed bars) relative to those in siControl (open bars) are indicated.

Interestingly, knockdown of ATF3 in LNCaP cells caused increase in p21 proteins (Fig. 6A) concomitant with reduction of cell growth (Fig. 6B). These findings are consistent with previous reports suggesting a role of ATF3 in cell proliferation in cancer[20], [22], [38] and c-Myc-induced cell growth[19]. In addition, current analysis of ATF3-regulated pathways in LNCaP cells (Table 3B) has indicated that as many as 13 genes in the p53 pathway might be activated by ATF3. To assess the effect of ATF3 on p53-mediated cell death, we carried out quantitative RT-PCR analysis of pro-apoptotic genes in p53 pathway. As illustrated in Fig. 6C, ATF3 knockdown caused up-regulation of FAS, TNFRSF10B/DR5, BBC3/PUMA, and CASP10 as well as CDKN1A/p21, suggesting that ATF3 negatively regulates p53-induced cell death and enhances cell proliferation through inhibition of p21 expression.

## Discussion

We showed that DNA damage-induced ATF3 binds to nearly one third of the human RefSeq promoters. Identification of such a large number of targets is not exceptional, since E2F1 and MYC are known to be recruited to >20,000 and >17,000 targets, respectively, in the human genome[39], and yeast INO4 has been reported to bind to 1,078 targets upon MMS stimulation[5]. The number of targets of cancer-associated ATF3 was smaller than that of DNA damage-induced ATF3, perhaps reflecting both higher levels and greater fold-induction of ATF3 in MMS response than those achieved by knockdown of ATF3 in LNCaP cells (data not shown). Nevertheless, the assay was remarkably consistent since 89% of targets in LNCaP cells were also found in MMS-treated HCT116 cells, and eleven out of 41 previously known ATF3 targets including EGR1[40] and HIF-2A[30] could be identified. A survey of ATF3 ChIP-seq data of the ENCODE database (http://hgdownload.cse.ucsc.edu/goldenPath/hg19/encodeDCC/wgEncodeHaibTfbs/) also indicates that significant proportions of ATF3 targets, i.e. 37.6% (768/2041) in K562 (leukaemia), 71.9% (742/1032) in HepG2 (liver), and 76.2% (809/1062) in H1-hESC (embryonic stem cells), are shared with 2711 targets in GM12878 (lymphoblastoid), which might be considered conservative figures given variations of ChIP-seq data quality (which affects the sensitivity of peak detection) and difference in tissue types.

Notably, bindings sites of several other transcription factors induced by DNA damage (NF-Y, E2F, YY1/NF-E1, FOXJ2, Sp1), UV irradiation (USF2, RFX1), and oxidative stress (c-Ets) were significantly over-represented in the ATF3 targets (Table 1) indicating an extensive network of these stress response factors (Fig. S6). A possible explanation for such coincidence of stress-related transcription factors is that ATF3 might synergize with these transcription factors to modulate target gene expression. Alternatively, ATF3 targets identified by ChIP analysis might include indirect binding of ATF3 through interaction with other stress-induced transcription factors. Indeed, ATF3 is a member of a large family of bZip type transcription factors, including those identified in the current study (i.e. AP-1, DDIT3, and USF2), which form homo- and hetero-dimers with different affinities to variants of the consensus ATF/CRE motif[32]. In addition, Sp1 found in the current analysis is known to physically interacts with ATF3[33], [34]. Further studies will be required to determine the role of stress-inducible transcription factors, especially those which has not been known to interact with ATF3 before, in binding of ATF3 to target genes.

The current study indicates that the impact of ATF3 on each target gene expression is not all-or-none effects. Rather, effects of ATF3 on many but select genes, such as those involved in cell cycle and cell death, collectively amounted to biologically significant outcome as cell death for ATF3 in stress response (Fig. 4C–E) or cell growth for cancer-associated ATF3(Fig. 6B). Moreover, our knockdown study shows that only 6–30% of the potential targets of ATF3 were directly regulated by ATF3 in line with previous reports showing that only 25% of p53 targets[6], 26% of STAT1 targets[41], and 11% of yeast transcription factor targets are affected by gene knockdown[5]. Several mechanisms have been proposed for p53 to explain why only a subset of potential targets respond to manipulations of p53 including epigenetic states of target genes, promoter occupancy with RNA polymerases, and recruitment of essential co-factors[42]. Similar mechanisms might be responsible for gene-selective effects of ATF3. Alternatively, multiple forms of ATF3 complexes, such as hetero-dimers with different bZip proteins, might underlie differential effects of ATF3 on target genes.

In the past several years, an increasing number of studies have indicated that ATF3 has pleiotropic functions depending on cell context. Importantly, our study revealed that stress-induced ATF3 and cancer-associated ATF3 have opposing effects on p53 and Wnt pathways: p53 pathway is activated in stress response (Fig. S5) consistent with the function of ATF3 in p53-mediated cell death [6], [23]–[25], whereas Wnt pathway is activated in cancer as previously reported in a study on ATF3 transgenic mice developing mammary tumours [43]. One might argue that the difference in genetic backgrounds and/or tissue types between HCT116 cells and LNCaP cells could have influenced the function of ATF3. Indeed, ATF3 appears to play distinct roles in different tissues: ATF3 acts as a tumour suppressor in colorectal cancer[24], [44] while it is oncogenic in prostate cancer[20], mammary cancer[21], skin cancer[29], and Hodgkin's lymphoma[22]. Our findings in HCT116 cells (colon) and LNCaP cells (prostate) are consistent with such a hypothesis.

Alterations of the regulatory function of ATF3 is highlighted by our finding that ATF3 is required for both activation and repression of pro-apoptotic genes such as BBC3/PUMA and TNFRSF10B/DR5 in stress response and cancer. Of note, opposing effects of ATF3 on cyclin D1 expression have been previously documented: ATF3 binds to AP-1 motif and activates cyclin D1 in mitogen-stimulated mouse hepatoma cells[45], whereas it binds to ATF/CRE site and represses cyclin D1 in mouse fibroblasts stimulated by serum[46]. In the literature, there are ample precedents of transcription factors which have context-dependent opposing functions on cancer development ([47] and reference therein). KLF4, for instance, activates p21 and represses p53 both of which are suppressed by activated Ras resulting in growth arrest in the absence of Ras or transforming phenotype in the presence of Ras. Alternatively, a recent study has shown that Kruppel-like factor 5 (KLF5) is required for MYC transcription in proliferating epithelial cells but is essential for TGFb-mediated repression of MYC[48]. Differential binding of KLF5 to TGFβ inhibitory element in the presence or absence of TGFβ was proposed as a mechanism of the opposite effects. Further studies will be required to determine if combinations of ATF3 and other transcription factors can switch the function of ATF3 on specific targets.

Resistance of tumour cells to various stress conditions remains an important issue. However, our knowledge about the role of cellular stress response machineries in cancer resistance to hypoxia[49] or chemotherapeutic agents[50] is still limited. Our finding that ATF3 has opposing effects on pro-apoptotic genes suggests that care must be taken to either enhance or block the function of ATF3 in a novel approach to cancer therapy. Further understanding of the switching mechanism of ATF3 function as a transcriptional activator or repressor might help develop strategies to selectively manipulate a subset of ATF3 target genes to assist the treatment of chemotherapy-resistant cancers.

## Materials and Methods

### Ethics statement

All animal work was approved and conducted according the guidelines of Committees of Animal Experiments and Recombinant DNA Experiments of Tokyo Medical and Dental University (License No. 2010-205).

### Plasmids

Luciferase reporters for DR5[51], GADD45A[52], PUMA[53], and vimentin[54] were kind gifts from Dr. Toshiyuki Sakai (Kyoto Prefectural University, Japan), Dr. Kazuhiro Daino (National Institute of Radiological Sciences, Japan), Dr. Jian Yu (The Johns Hopkins Oncology Center, USA), and Dr. Susan Rittling (The Forsyth Institute, USA), respectively. The DR5 luciferase reporter was reconstructed by subcloning a DR promoter fragment from PstI (−1,226) to the AUG codon into PicaGene PGV-B2 vector (Toyo B-Net Co., Ltd, Japan). The pCI-ATF3 expression vector was described previously[55].

### Cell culture

Human colorectal carcinoma HCT116 cells and prostate carcinoma LNCaP cells were obtained from American Type Culture Collection (USA). Forty-eight hours before stimulation by MMS, culture medium of HCT116 cells were replaced with the medium containing 0.25% FCS. Twenty-four hours later, HCT116 cells were transfected with either control siRNA or siATF3 by using X-tremeGENE siRNA Transfection Reagent. ON-TARGETplus siRNA SMART pool from Dharmacon was used for knockdown of ATF3. After 24 hours, MMS was added to final concentration of 50 ng/ml to HCT116 cells and cultured for 0, 6, 12, and 24 hours.

### Luciferase assay

Mouse embryonic fibroblasts were obtained from wildtype or ATF3^-/-^ embryos at 13.5 d.p.c. Luciferase reporters and ATF3 expression vectors were co-transfected into HCT116 cells or mouse embryonic fibroblasts, stimulated with 50 ng/ml MMS for 12 hours, and whole cell extracts were analysed by Dual Luciferase Reporter Assay System (Promega) using Lumat LB 9507 luminometer (Berthold Japan).

### ChIP-chip analysis

Chromatin immunoprecipitation of HCT116 cells and LNCaP cells was performed by using anti-ATF3 rabbit polyclonal antibodies (Santa Cruz) and ChIP-IT Chromatin Immunoprecipitation Kit (Active Motif). DNA was fragmented to less than 500 bp in length by sonication with BioRuptor (Cosmo bio), and hybridized to NimbleGen Human ChIP 385K RefSeq Promoter Arrays (Roche). MA2C package[31] was then used for normalization and peak detection with 500 bp window, scoring by trimmed mean, lower-bound of false discovery rate at 0.2%, and default settings for other parameters. The ChIP and expression profiling data were assigned GEO accession number GSE18457.

### Expression profiling and pathway mapping

Total RNA was extracted from MMS-treated HCT116 cells, labelled with Cy3, and hybridized to Whole Human Genome Microarray Kit, 4×44K (Agilent). Normalization between arrays was done by average signals of seven house keeping genes (RPS13, RPL27, RPS20, RPL30, RPL13A, RPL9, and SRP14)[56]. Gene lists either up- (ratio >1.2) or down-regulated (ratio <0.8) were subjected to pathway mapping using DAVID[35]. Quantitative RT-PCR was carried out using SYBR Green PCR Master Mix and 7900HT Real Time PCR System (Applied Biosystems).

### TRANSFAC motif scan

Transcription factor binding sites over-represented in ATF3 targets were searched by ExPlain v.2.4.1 analysis system and its F-Match algorithm (BIOBASE GmbH). Input lists of ATF3-target genes in MMS-treated HCT116 cells and LNCaP cells and of non-ATF3 target genes (background) were prepared as UniGene ID for matrix searching with the following parameters: matrix profile of vertebrate_non_redundant (minSUM); cut-offs of minSUM; promoter window parameter from -1000 to 100 around the TSS; multiple promoter parameter of all promoters.

## Supporting Information

Figure S1 **Quality control of ChIP-on-chip analysis.** (A) Left: Ethidium bromide staining of genomic DNA of MMS-treated HCT116 cells from whole cell extract (WCE) or from chromatin immunoprecipitated with anti-ATF3 antibodies. Right: FDR table of MA2C analysis[31] indicating predicted number of peaks at different FDR values. (B) Left: Ethidium bromide staining of genomic DNA of LNCaP cells from whole cell extract or from chromatin immunoprecipitated with anti-ATF3 antibodies. Right: FDR table of MA2C analysis indicating predicted number of peaks at different FDR values.
(TIF)

Figure S2 **TRANSFAC motif scan of ATF3 targets.** ATF3 targets unique to MMS-treated HCT116 cells or LNCaP cells and those common between them were scanned against TRANSFAC database along with a background gene set. The number of hits of each motif in ATF3 targets (hashed bars) or a background gene set (open bars) is shown.
(TIF)

Figure S3 **Effect of ATF3 knockdown on gene expression in DNA damage response.** (A) Normalization of signals of different arrays using seven house keeping genes [56]. (B) Distribution of signals in control cells (blue) and ATF3 knockdown cells (red). Peaks in the far left of each histogram reflect those which are not expressed above background levels. (C) Summary of average signal ratio (log_2_ values) between ATF3 knockdown and control cells showing that ATF3 knockdown causes minimal changes in average gene expression levels.
(TIF)

Figure S4 **Effect of ATF3 knockdown on gene expression in LNCaP cells.** Distribution of signals in LNCaP cells transfected with siControl (top panel) or siATF3 (bottom panel). Peaks in the far left of each histogram reflect those which are not expressed above background levels.
(TIF)

Figure S5 **Transcriptional co-regulation of p53 pathway by ATF3.** Effect of ATF3 on expression of select p53 targets which are involved in regulation of apoptosis, DNA repair, mTOR, cell cycle, transcription, Wnt pathway, adhesion, and endosome function. Genes activated or repressed by ATF3 are coloured red or green, respectively.
(TIF)

Figure S6 **Transcriptional co-regulatory network of stress responses.** ATF3 is a hub of an extensively overlapping network of stress sensors (solid lines) which enables cells to respond to various stress signals (dotted lines). Epistatic regulations (arrow heads) and functional interactions (dot ends) are indicated.
(TIF)
