# Supplementary figures and images for: Systems Analysis of ATF3 in Stress Response and Cancer Reveals Opposing Effects on Pro-Apoptotic Genes in p53 Pathway
Source: PLoS One. 2011 Oct 26;6(10):e26848. doi: 10.1371/journal.pone.0026848 (PMC3202577; doi:10.1371/journal.pone.0026848)

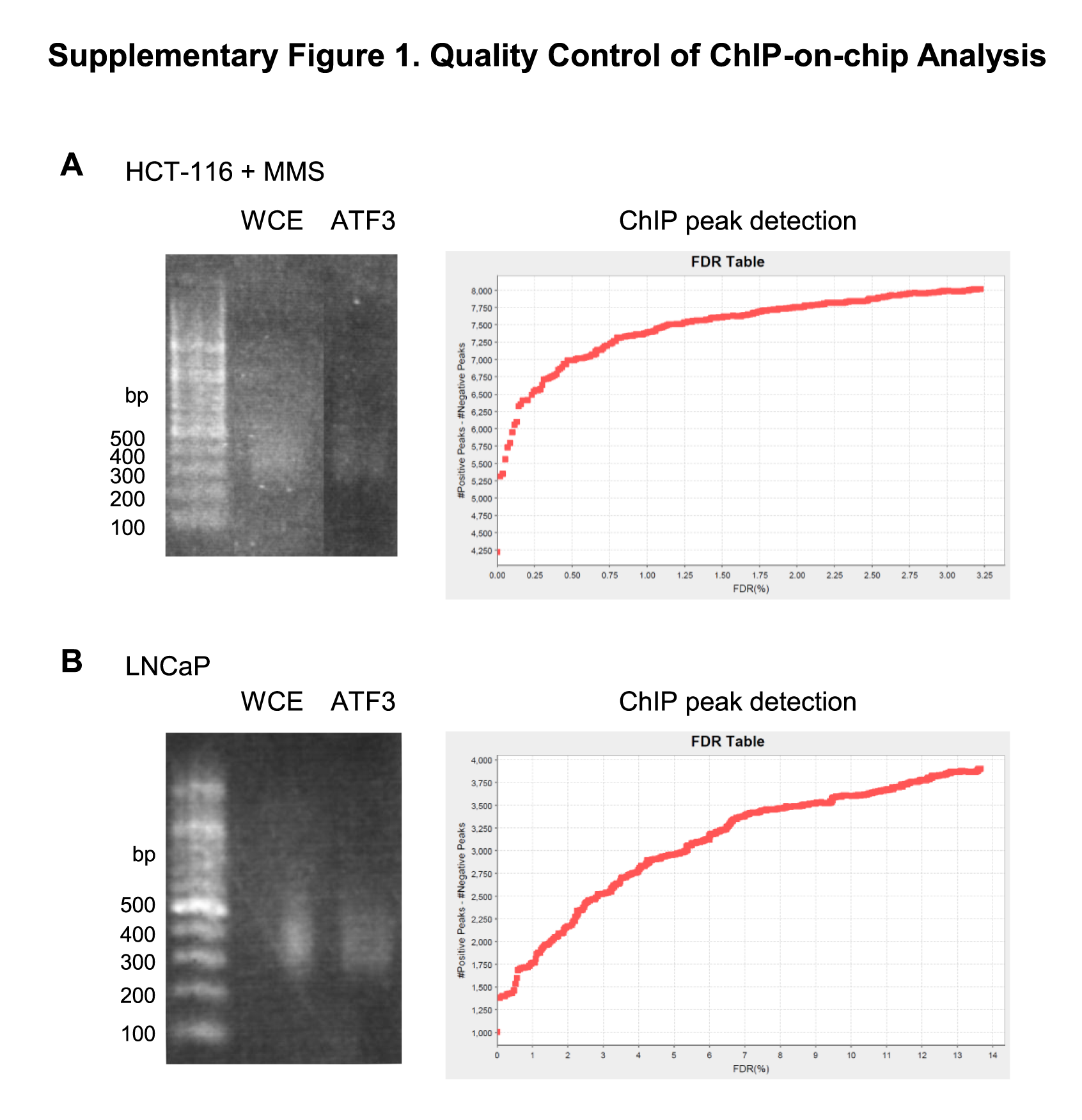

Supplement: Figure S1 — Quality control of ChIP-on-chip analysis. (A) Left: Ethidium bromide staining of genomic DNA of MMS-treated HCT116 cells from whole cell extract (WCE) or from chromatin immunoprecipitated with anti-ATF3 antibodies. Right: FDR table of MA2C analysis[31] indicating predicted number of peaks at different FDR values. (B) Left: Ethidium bromide staining of genomic DNA of LNCaP cells from whole cell extract or from chromatin immunoprecipitated with anti-ATF3 antibodies. Right: FDR table of MA2C analysis indicating predicted number of peaks at different FDR values. (TIF) [file pone.0026848.s001.tif]

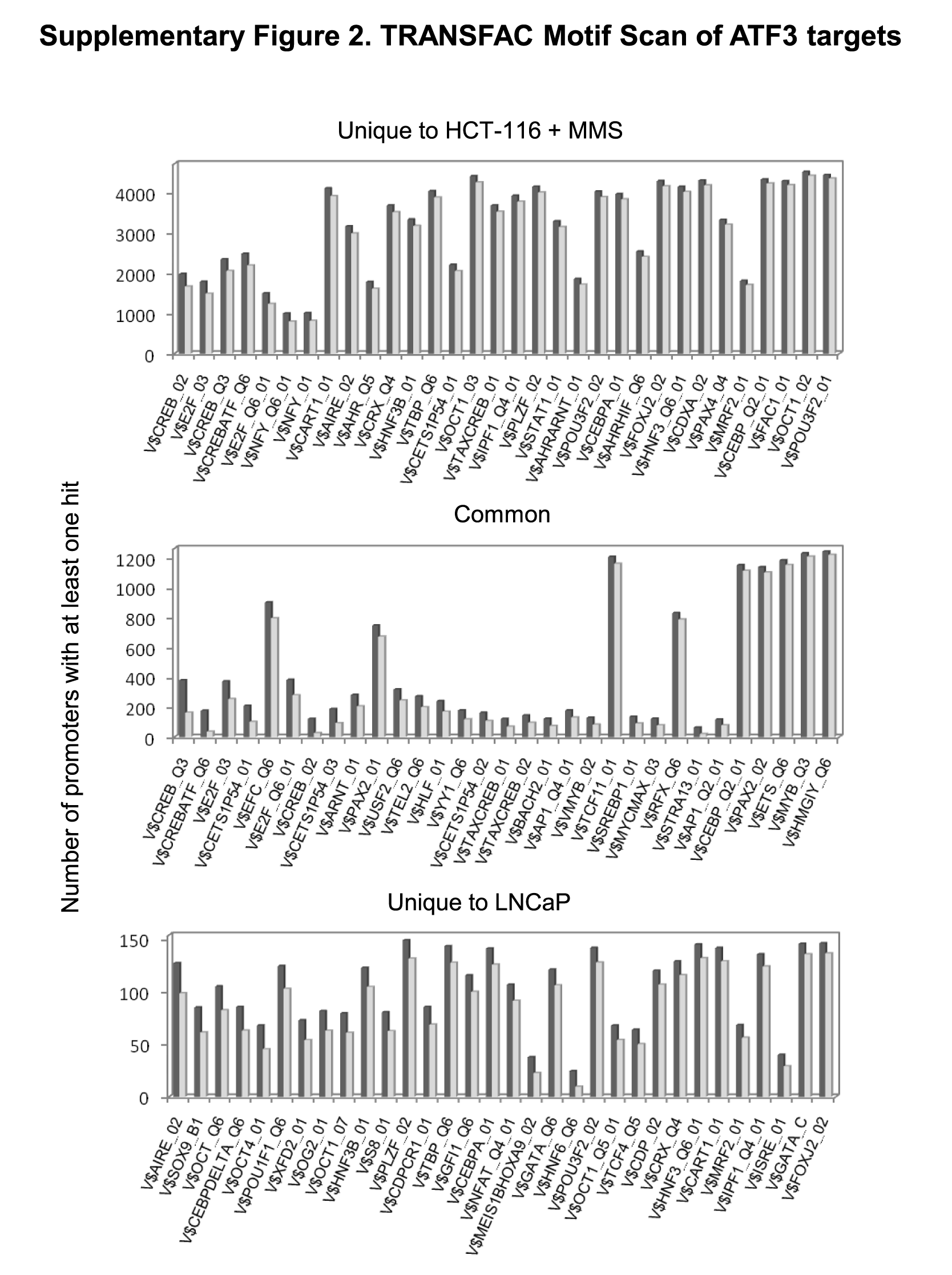

Supplement: Figure S2 — TRANSFAC motif scan of ATF3 targets. ATF3 targets unique to MMS-treated HCT116 cells or LNCaP cells and those common between them were scanned against TRANSFAC database along with a background gene set. The number of hits of each motif in ATF3 targets (hashed bars) or a background gene set (open bars) is shown. (TIF) [file pone.0026848.s002.tif]

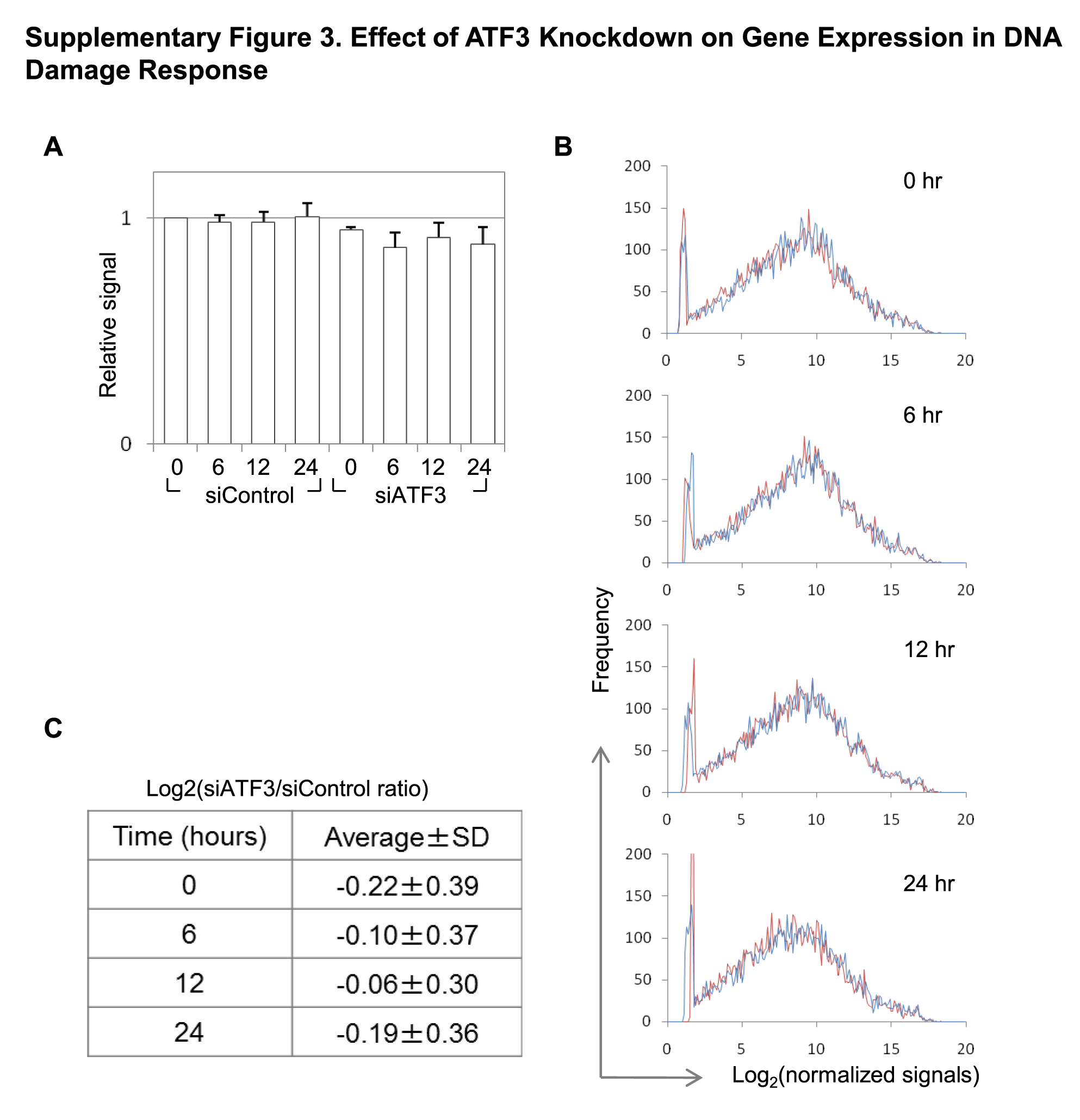

Supplement: Figure S3 — Effect of ATF3 knockdown on gene expression in DNA damage response. (A) Normalization of signals of different arrays using seven house keeping genes [56]. (B) Distribution of signals in control cells (blue) and ATF3 knockdown cells (red). Peaks in the far left of each histogram reflect those which are not expressed above background levels. (C) Summary of average signal ratio (log2 values) between ATF3 knockdown and control cells showing that ATF3 knockdown causes minimal changes in average gene expression levels. (TIF) [file pone.0026848.s003.tif]

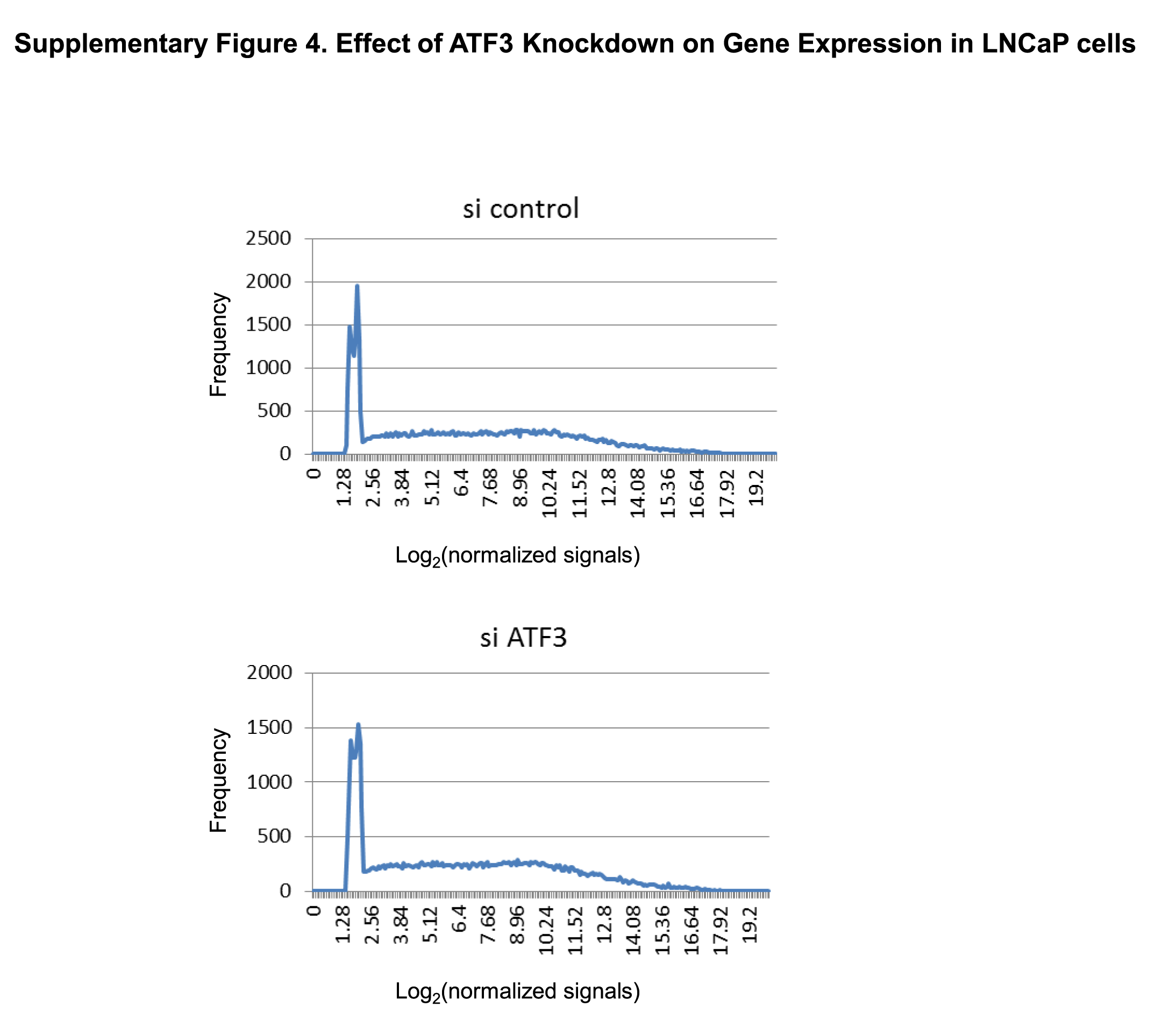

Supplement: Figure S4 — Effect of ATF3 knockdown on gene expression in LNCaP cells. Distribution of signals in LNCaP cells transfected with siControl (top panel) or siATF3 (bottom panel). Peaks in the far left of each histogram reflect those which are not expressed above background levels. (TIF) [file pone.0026848.s004.tif]

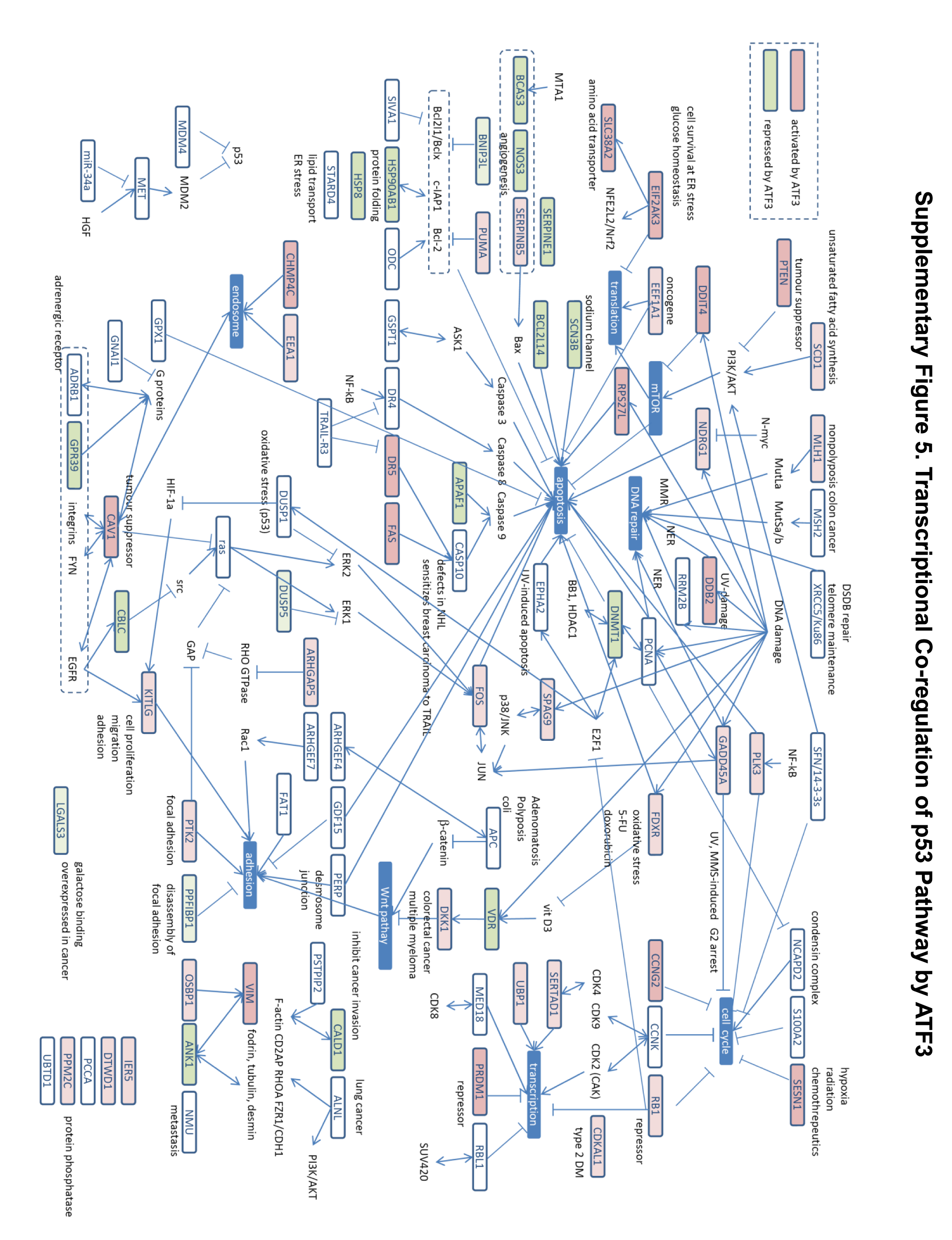

Supplement: Figure S5 — Transcriptional co-regulation of p53 pathway by ATF3. Effect of ATF3 on expression of select p53 targets which are involved in regulation of apoptosis, DNA repair, mTOR, cell cycle, transcription, Wnt pathway, adhesion, and endosome function. Genes activated or repressed by ATF3 are coloured red or green, respectively. (TIF) [file pone.0026848.s005.tif]

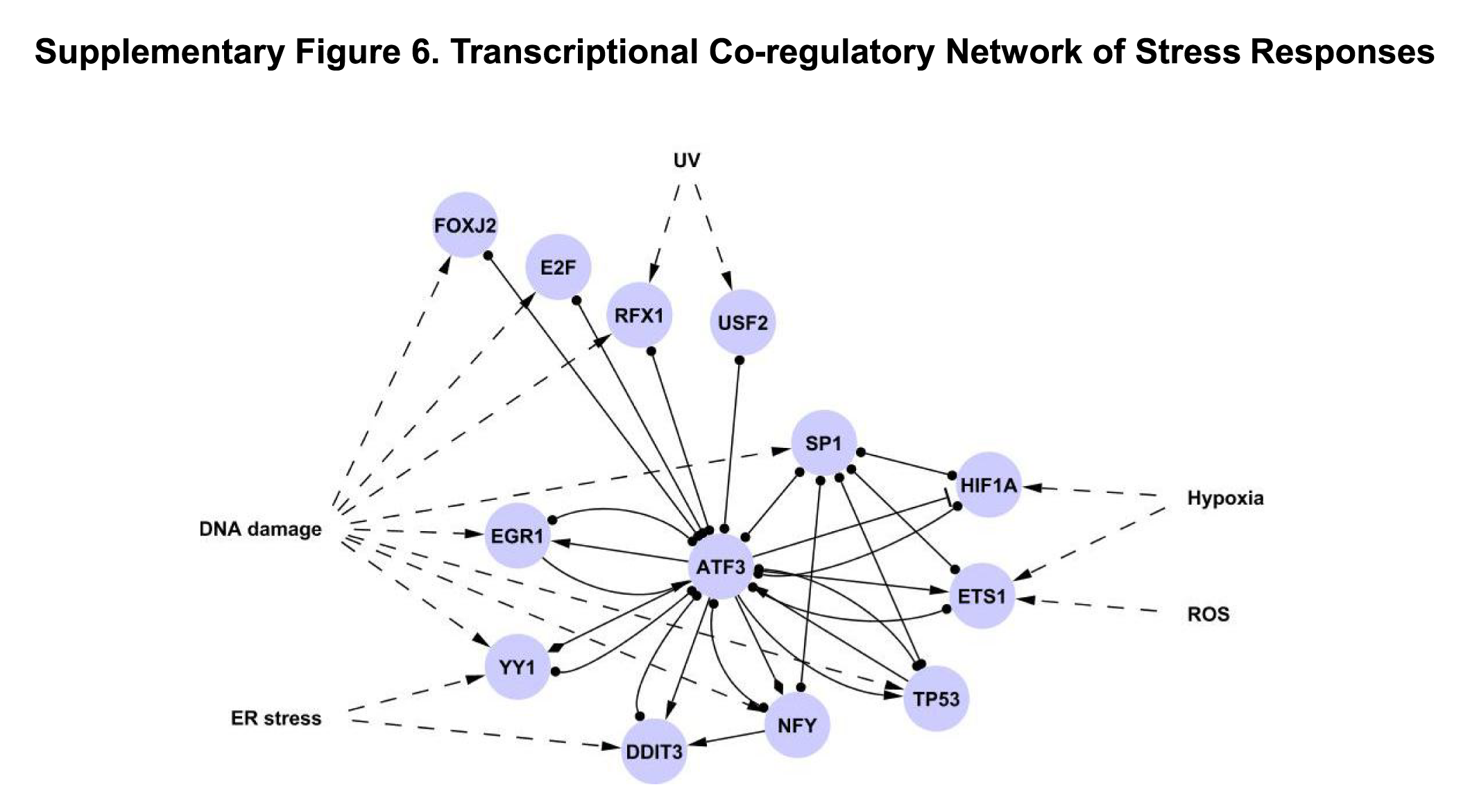

Supplement: Figure S6 — Transcriptional co-regulatory network of stress responses. ATF3 is a hub of an extensively overlapping network of stress sensors (solid lines) which enables cells to respond to various stress signals (dotted lines). Epistatic regulations (arrow heads) and functional interactions (dot ends) are indicated. (TIF) [file pone.0026848.s006.tif]
